# Influencing factors of alexithymia in Chinese medical students: a cross-sectional study

**DOI:** 10.1186/s12909-017-0901-8

**Published:** 2017-04-04

**Authors:** Yaxin Zhu, Ting Luo, Jie Liu, Bo Qu

**Affiliations:** 1 School of Public Health, China Medical University, Shenyang, Liaoning Province People’s Republic of China; 2 Research Center for Medical Education, China Medical University, No. 77 Puhe Road, Shenyang North New Area, Shenyang, Liaoning Province Postal Code: 110122 People’s Republic of China

**Keywords:** Alexithymia, Medical students, 20-item Toronto Alexithymia Scale, Influencing factors

## Abstract

**Background:**

A much higher prevalence of alexithymia has been reported in medical students compared with the general population, and alexithymia is a risk factor that increases vulnerability to mental disorders. Our aim was to evaluate the level of alexithymia in Chinese medical students and to explore its influencing factors.

**Methods:**

A cross-sectional study of 1,950 medical students at Shenyang Medical College was conducted in May 2014 to evaluate alexithymia in medical students using the Chinese version of the 20-item Toronto Alexithymia Scale (TAS-20). The reliability of the questionnaire was assessed by Cronbach’s α coefficient and mean inter-item correlations. Confirmatory factor analysis (CFA) was used to evaluate construct validity. The relationships between alexithymia and influencing factors were examined using Student’s *t*-test, analysis of variance, and multiple linear regression analysis. Statistical analysis was performed using SPSS 21.0.

**Results:**

Of the 1,950 medical students, 1,886 (96.7%) completed questionnaires. Overall, Cronbach’s α coefficient of the TAS-20 questionnaire was 0.868. The results of CFA showed that the original three-factor structure produced an acceptable fit to the data. By univariate analysis, gender, grade (academic year of study), smoking behavior, alcohol use, physical activity, history of living with parents during childhood, and childhood trauma were influencing factors of TAS-20 scores (*p* < 0.05). Multiple linear regression analysis showed that gender, physical activity, grade, living with parents, and childhood trauma also had statistically significant association with total TAS-20 score (*p* < 0.05).

**Conclusions:**

Gender, physical activity, grade, history of living with parents during childhood, and childhood trauma were all factors determining the level of alexithymia. To prevent alexithymia, it will be advisable to promote adequate physical activity and pay greater attention to male medical students and those who are in the final year of training.

**Electronic supplementary material:**

The online version of this article (doi:10.1186/s12909-017-0901-8) contains supplementary material, which is available to authorized users.

## Background

With the remarkable improvement in living standards in China, people are more conscious of their health and expect higher-quality medical services [1]. And physicians are sometimes unable to meet patient demands for satisfactory medical services [2]. The resulting gap between patient expectations and physician objectives has generated a deterioration of the doctor-patient relationship over the past decade [3, 4]. This strained relationship combined with poor doctor-patient communication has sometimes resulted in medical errors, malpractice litigation, and negative patient experience [5, 6]. The needs of patients is the central focus of the doctors’ work, which might be inherently emotionally demanding [7]. Thus there is a critical need for doctors to put emotion into their work, which could improve the quality of health care service and doctor-patient relationship [8, 9].

One barrier to a satisfying doctor-patient relationship is the inability of a physician to be aware of, and subsequently being able to modulate and manage, emotions in themselves and others, a personality construct known as alexithymia [10]. The term alexithymia was first defined by Sifneos as meaning “no words for feeling”. This multidimensional construct is used in psychiatry and psychosomatic medicine to reflect a cognitive and affective style marked by difficulties in verbally describing affection and in differentiating mental states from bodily sensations; a paucity of fantasy; and utilitarian thinking [11, 12].

A recent study indicated that much of professional alexithymia originated during the medical education program, and suggested that the emotional dimensions of daily interactions between medical students and others deserved attention [10]. A much higher prevalence of alexithymia has been reported in medical students compared with the general population [13, 14]. Due to the demands of medical education, including work overload, frequent examinations, and a competitive environment, medical students confront an array of intense emotions and are at high risk of developing mental disorders such as anxiety [15], depression [16], and burnout [17]. Alexithymia is a risk factor that increases vulnerability to these same disorders [18–21]. Thus, the level of alexithymia among medical students is of great concern and signals the necessity for a higher level of focus on this problem.

It has been suggested that approaching the problem of alexithymia in medical students by assessing and addressing risk factors should become an issue at medical universities [13]. Several factors associated with alexithymia have been described. One study conducted with Romanian medical students showed that male subjects were more affected than females [13]. Some evidence indicates that adolescents who reported growing up with less parental care or who experienced childhood trauma were more prone to exhibit alexithymic features [22, 23]. Helmers and Mente proposed that alcohol use and a sedentary lifestyle with little physical exercise were correlated with difficulty in communicating feelings [24]. A longitudinal study also revealed that alexithymia was related to the level of physical activity in adolescent females [25].

A number of researchers have introduced instruments to measure alexithymia, including the Observer-Alexithymia Scale [26], the Toronto Structured Interview for Alexithymia (TSIA) [27], and the 20-item Toronto Alexithymia Scale (TAS-20) [14, 28, 29]. The Observer-Alexithymia Scale and TSIA are observer-based measurements that have a potential weakness in the capacity to produce reliable ratings of respondent awareness across different interviewers and other raters [27]. The TAS-20 is a multidimensional self-reported instrument with a three-factor structure that accurately assesses three salient facets of the alexithymia construct, including difficulty identifying feelings (DIF), difficulty describing feelings (DDF), and externally oriented thinking (EOT) [30]. It has been widely used to assess alexithymia in different languages and cultures, including Dutch [28], Japanese [29], and German [14]. The Chinese version was developed by Yi [31, 32]. The original English version of the TAS-20 was first translated into Chinese and then back translated and modified until cross-culture equivalence was established [32]. The reliability and validity of the Chinese version of the TAS-20 have been well demonstrated in the study involving medical students and clinical patients [32].

The purpose of the present study is to evaluate the level of alexithymia and to explore its influencing factors among Chinese medical students, in order to provide references for prevention strategies on alexithymia.

## Methods

### Ethics statement

Only medical students who gave written informed consent were enrolled in the study. The investigator informed all participants about the purpose of study and assured them of anonymity before research began. The protocol was approved by the Bioethics Advisory Commission of China medical university.

### Participants and procedures

Medical training of a Chinese medical college lasts 5 years and is divided into 2 years of basic sciences, 2 years of clinical medicine, and 1 year of internship. Medical students are admitted directly from high school and they can take part in the national entrance examination to pursue medical postgraduate studies after their graduation. The present study was conducted in May, 2014 and recruited 1,950 medical students from five grades at Shenyang Medical College. Subjects were selected using method with stratified-cluster random sampling. Medical students were stratified by the grade. Several classes were extracted proportionally to the class ratio per grade level, and all students from these selected classes participated in our study.

### Instruments

Students voluntarily completed self-administered questionnaires. The survey questionnaire comprised two sections including basic information and the TAS-20. The basic information form included socio-demographic information (age, gender, body mass index [BMI], residence, and grade), as well as life-related characteristics (alcohol use, smoking status, physical activity, history of living with parents during childhood, and experience of childhood trauma). The basic information data were collected using a self-designed questionnaire consisting of some items. The item to assess the level of physical activity was that “How often do you take part in physical training”. And the three possible answers to this item were several times a week, less than once a week, and never. One item “Have you ever experienced emotional, physical, or sexual abuse during childhood” in a “yes or no” format was used to evaluate the childhood trauma.

The TAS-20 [30] is a self-reported instrument rated on a 5-point Likert-type scale ranging from 1 (strongly disagree) to 5 (strongly agree). The TAS-20 encompasses three subscales: DIF, DDF and EOT, each yielding a subscale score ranging from 7 to 35, 5 to 25, and 8 to 40, respectively. Total TAS-20 score range from 20 to 100, with higher scores demonstrating higher levels of alexithymia. In traditional cutoffs of the TAS-20 scale, total score >60 indicate having alexithymia [33]. The form of TAS-20 instrument is included in Additional file 1.

### Statistical analysis

Cronbach’s α coefficient and mean interitem correlations (MICs) were calculated to evaluate the internal consistency reliability and item-to-item homogeneity of the TAS-20. Confirmatory factor analysis (CFA) was performed to test the construct validity of the TAS-20 in the current sample. Goodness of fit was evaluated using the indexes including chi-square (*χ* ^*2*^), root mean square error of approximation (RMSEA), comparative fit index (CFI), and adjusted goodness of fit index (AGFI). A RMSEA value below 0.08 and a CFI value above 0.90 indicated a good fit [34]. For AGFI, a value above 0.85 was commonly considered adequate model fit [34]. Student’s *t*-test and one-way analysis of variance (ANOVA) followed by post-hoc LSD tests were performed to test the association between variables in socio-demographic data, life-related factors, and TAS-20 scores. To assess the influence of different factors on alexithymia, we performed a multiple linear regression analysis with the total score of TAS-20 as dependent variable. And the significant variables in the results of univariate analysis were independent variables. All significant variables in the univariate analysis were then entered into a multiple linear regression model, and stepwise method was used. The data were analyzed using SPSS version 21.0 (SPSS Inc., Chicago, IL, USA) for Windows. A *p*-value of < 0.05 was considered to be statistically significant.

## Results

### Socio-demographic characteristics of medical students

Of the 1,950 medical students, 1,886 completed the questionnaires, with a response rate of 96.7%. The socio-demographic characteristics of the participants are described in Table 1. There were 1,518 female (80.5%) and 368 (19.5%) male participants. The ages ranged from 17 to 25 years, with a mean of 20.6 years (SD = 1.67). Over half of students were from rural areas (*n* = 1,017, 53.5%). The average BMI was 20.79 kg · m^−2^ (SD = 2.97). In addition, sport activities including running (46.4%) and ballgames (24.9%) were more popular among the participants compared with yoga (13.1%), dancing (10.3%), or swimming (5.3%). The mean total TAS-20 score was 51.55 (SD = 9.12). Using the traditional TAS-20 cutoff score of >60, 297 (15.7%) participants were considered alexithymic. The prevalence of alexithymia was higher among male medical students (88/368, 23.9%) than among females (209/1,518, 13.8%).

**Table 1:** Univariate analysis on influencing factors of TAS-20 scores among medical students (*n* = 1886)

Variable	N (%)	DIF	DDF	EOT	TAS-20
Total participants	1,886 (NA)	17.49 ± 4.87	13.29 ± 3.12	20.78 ± 3.35	51.55 ± 9.12
Gender^a^					
Male	368 (19.5%)	**18.60 ± 5.20** ^*^	**13.81 ± 3.17** ^*^	**21.38 ± 3.19** ^*^	**53.79 ± 9.39** ^*^
Female	1,518 (80.5%)	**17.22 ± 4.75**	**13.16 ± 3.09**	**20.63 ± 3.37**	**51.01 ± 8.98**
Grade^b^					
One^c^	584 (31.0%)	**17.01 ± 4.65** ^*^	13.05 ± 3.14	**20.39 ± 3.61** ^*^	**50.45 ± 8.65** ^*^
Two^c^	473 (25.1%)	**17.14 ± 4.82**	13.37 ± 3.08	**20.56 ± 3.40**	**51.67 ± 9.05**
Three^c^	361 (19.1%)	**17.53 ± 5.05**	13.29 ± 3.06	**20.79 ± 3.46**	**51.61 ± 9.18**
Four^c^	276 (14.6%)	**17.18 ± 4.73**	13.32 ± 3.13	**20.93 ± 3.28**	**51.42 ± 9.08**
Five	192 (10.2%)	**18.68 ± 5.32** ^**a**^	13.75 ± 3.19	**22.20 ± 3.25** ^**a**^	**54.66 ± 9.05** ^**a**^
Residence^a^					
Urban	869 (46.1%)	17.39 ± 5.22	**13.03 ± 3.30** ^*^	20.74 ± 3.46	51.16 ± 9.75
Rural	1,017 (53.9%)	17.57 ± 4.56	**13.50 ± 3.30**	20.80 ± 3.25	51.88 ± 8.55
BMI^b^					
< =18.4	317 (16.8%)	17.13 **±** 4.46	13.13 ± 2.96	20.59 ± 3.15	50.85 ± 8.48
18.5–24.9	1,412 (74.9%)	17.56 **±** 4.92	13.33 ± 3.14	20.83 ± 3.35	51.71 ± 9.21
> =25	157 (8.3%)	17.57 **±** 5.24	13.20 ± 3.20	20.69 ± 3.70	51.46 ± 9.60
Alcohol use^a^					
Yes	226 (12.0%)	**18.86 ± 5.45** ^*^	13.57 ± 3.05	20.73 ± 3.47	**53.16 ± 10.26** ^*^
No	1,660 (80.0%)	**17.30 ± 4.76**	13.25 ± 3.50	20.78 ± 3.33	**51.33 ± 8.94**
Smoking behavior^a^					
Yes	60 (3.2%)	**19.33 ± 6.00** ^*^	13.85 ± 3.67	21.48 ± 4.08	**54.67 ± 11.68** ^*^
No	1,826 (96.8%)	**17.43 ± 4.82**	13.27 ± 3.10	20.75 ± 3.32	**51.45 ± 9.01**
Living with parents^a^					
Yes	1,674 (88.8%)	**17.27 ± 4.85** ^*^	**13.17 ± 3.10** ^*^	20.73 ± 3.32	**51.18 ± 9.13** ^*^
No	212 (11.2%)	**19.17 ± 4.75**	**14.21 ± 3.08**	21.11 ± 3.52	**54.49 ± 8.49**
Childhood trauma^a^					
Yes	123 (6.5%)	**19.40 ± 5.47** ^*^	**14.02 ± 3.85** ^*^	20.93 ± 3.59	**54.35 ± 10.96** ^*^
No	1,763 (93.5%)	**17.35 ± 4.80**	**13.23 ± 3.05**	20.76 ± 3.33	**51.35 ± 8.95**
Physical activity^b^					
Never	172 (9.1%)	**18.40 ± 5.12** ^*^	**14.14 ± 3.14** ^*^	**21.62 ± 3.33** ^*^	**54.16 ± 9.27** ^*^
Less than once a week^d^	1,522 (80.7%)	**17.59 ± 4.74**	**13.23 ± 3.07**	**20.74 ± 3.31**	**51.33 ± 8.90**
Several times a week	192 (10.2%)	**17.37 ± 5.59** ^**b**^	**12.99 ± 3.37** ^**b**^	**20.33 ± 3.50** ^**b**^	**50.91 ± 10.33** ^**b**^

*DIF* Difficulty identifying feelings, *DDF* Difficulty describing feelings, *EOT* Externally oriented thinking, *TAS-20* 20-item Toronto Alexithymia Scale

*Statistically significant associations are bolded (*p* < 0.05)

^a^Student’s *t*-test; ^b^One-way ANOVA

^c^Significantly different compared with grades one, two, three, and four, respectively (*p* < 0.05 at DIF, EOT, and TAS-20)

^d^Significantly different compared with groups less than once a week and never, respectively (*p* < 0.05 at DIF, DDF, EOT, and TAS-20)

### Reliability and validity analysis

The internal consistency reliability for the TAS-20 had an overall Cronbach’s α coefficient of 0.87. All 3 subscales were confirmed adequate internal consistency, with the an alpha level ranging from 0.72 (DDF) to 0.83 (DIF). The MIC values for total TAS-20, DIF, DDF, and EOT were 0.26, 0.40, 0.25, and 0.16, respectively. The results of CFA showed that the three-factor structure of the TAS-20 produced an acceptable fit to the data (*χ* ^2^ = 2098.75, df = 167, *P* < 0.001; CFI = 0.88; RMSEA = 0.076 (90% CI: 0.073–0.079); AGFI = 0.85).

### Exploratory investigation of possible related factors for alexithymia among medical students

In the univariate factor analysis shown in Table 1, there were significant differences in scores for total TAS-20 and all three subscales between different groups of gender and physical activity (*p* < 0.05). Compared with female, the male students got higher scores. And the medical students who participated in the physical activity several times a week had the lowest scores. Total TAS-20, DIF, and EOT scores showed significant grade differences (*p* < 0.05), and the fifth grade students had the highest scores. The significant differences were also found in total TAS-20, DIF, and DDF scores between different groups of living with parents during childhood and childhood trauma (*p* < 0.05). And participants who lived without parents during childhood and had childhood trauma had higher scores. In addition, the total TAS-20 and DIF scores were significantly higher in the medical students who smoked and consumed alcohol (*p* < 0.05). No statistically significant difference of TAS-20 scores were detected between different BMI groups.

The results of multiple linear regression analysis are shown in Table 2. There were five factors included in the regression model. Gender, childhood trauma, and physical activity were negatively correlated with the total score of TAS-20 (*p* < 0.05). Gender, childhood trauma, and physical activity had the standardized coefficient of −0.116, −0.070, and −0.082, respectively. Grade and history of living with parents during childhood were positively associated with total TAS-20 score among medical student (*p* < 0.05).

**Table 2:** Results of multiple linear regression analysis for influencing factors of total TAS-20 score (*n* = 1,886)

Factor	Unstandardized coefficient		Standardized coefficient	t	p
	B	SE	β		
Gender^a^	−2.670	0.530	−0.116	−5.035	0.000
Grade^b^	0.503	0.158	0.073	3.191	0.001
Living with parents^c^	3.068	0.652	0.106	4.706	0.000
Childhood trauma^d^	−2.602	0.834	−0.070	−3.119	0.002
Physical activity^e^	−1.696	0.481	−0.082	−3.529	0.000

*TAS-20* 20-item Toronto Alexithymia Scale; *B* Regression coefficient, *SE* Standardized error

^a^Male = 1; Female = 2

^b^Grade one = 1; Grade two = 2; Grade three = 3; Grade four = 4; Grade five = 5

^c^Yes = 1; No = 2

^d^Yes = 1; No = 2

^e^Never = 1; Less than once a week = 2; Several times a week = 3

## Discussion

The present study confirmed that the Chinese version of the TAS-20 was reliable and valid to measure alexithymia among medical students. The results of multiple linear regression analysis revealed that gender, physical activity, grade, history of living with parents, and childhood trauma had significant association with total TAS-20 score (*p* < 0.05).

The results of this study showed acceptable psychometric properties of the Chinese version of the TAS-20 in a large sample of medical students. Cronbach’s α coefficient and the MIC for the total TAS-20 scale were 0.87 and 0.26, respectively, indicating adequate internal consistency and acceptable homogeneity [35, 36]. The original three-factor structure of the TAS-20 produced an acceptable fit to our data, which is similar to the findings of Turkish medical students [37].

The results of the multiple linear regression analysis demonstrated that male gender was significantly associated with higher level of alexithymia (*p* < 0.05). Studies on college students indicate that a higher level of alexithymia is typically exhibited by males rather than females [13, 38]. Compared with women, who typically have more emotion regulation strategies for managing attendant emotional states, men do not develop much verbal expression of their emotions [39]. This gender-linked characteristic has been explained by the Normative Male Alexithymia (NMA) hypothesis, which describes the inability of men to verbally express their emotions as a result of traditional masculine gender role socialization [40]. According to this view of traditional masculinity ideology, males do not develop a vocabulary for negative emotions such as stress or anxiety that might make them appear weak or dependent [41]. It has been proposed that men with higher levels of NMA are less likely to communicate effectively with their partners and experience more difficulty developing healthy and intimate social relationships [42].

Our study also indicates that medical students who never engage in physical activity exhibited higher scores for TAS-20 compared with those who frequently or only occasionally exercise (*p* < 0.05). This result is in agreement with previous findings that inadequate physical activity significantly correlated with higher levels of alexithymia [24, 25]. This could be explained by improved cardiovascular flexibility and neurocognitive functioning, which have been implicated in the modulation and processing of emotions, respectively [43–46]. Helmers and Mente also found that poor exercise, as a result of a sedentary lifestyle, may lead to difficulty in communicating feelings and expressing emotions [24]. Due to the large number of hours required for medical curriculum, medical students have too little time for regular physical activity, and consequently develop a physically inactive lifestyle [47]. We also found that running and ballgames enjoy the widest popularity among medical students. Therefore, it is suggested that educators and administrators include sufficient time for exercise in medical university programs, and that they ensure superior sports facilities for running and ballgames, in particular.

It was noteworthy that medical students in 50 year exhibited the highest scores for TAS-20 (*p* < 0.05). In the final college year practicing in hospitals, medical students may face even more stress due to the increased complexity of the interpersonal environment and a lack of adequate clinical skills [48]. With a strong desire to promote individual core competence and developmental space, 50 year medical students who plan to pursue postgraduate training are also under great stress from increasing competition [49]. In addition, medical students face increasing employment pressure due to uncertain employment prospects in the current Chinese labor market [50]. Hua et al. suggested that a hyper-responsive hypothalamo-pituitary-adrenal system during stress is related to difficulty in differentiating feelings and distinguishing them from bodily sensations and emotional arousal [51]. In another study, alexithymics under tremendous stress tended to limit expression of their stressful emotional experience by employing an avoidance-oriented coping style, and their undifferentiated emotions were perpetuated, eventually strengthening the alexithymic state [52].

Our survey showed that medical students who lived with parents during childhood were associated with lower score for total TAS-20 (*p* < 0.05). This was consistent with research on British student populations that supported the crucial role of parents in the development of emotional behaviors [53]. It is thought that people develop the ability to experience their own and other people’s feelings on a verbal and mental level with help from their parents, who accurately sense, identify, and name the relevant affects [54]. Repeated parental unresponsiveness and parental absence might exert negative effects on the organization of emotion schemes and the construction of emotional meanings, and thereby favor the gradual development of alexithymic characteristics [55, 56].

Our research also found that medical students who experienced childhood trauma were associated with higher score for total TAS-20 (*p* < 0.05). This was consistent with clinical observations proposing that childhood trauma may have an impact on the development of alexithymia [57]. Psychic trauma during early childhood may influence the capacity to effectively self-regulate affective states, thereby leading to alexithymia [58].

This study had some limitations. The cross-sectional nature of data limits our findings to associations only, and precludes conclusions regarding causality. The sample was drawn from only one college, and the female-to-male ratio was a little higher than other medical colleges in China. So the study population is not demographically diverse enough to be representative of all Chinese medical students. And further research could improve representativeness of the sample by expanding sample diversity.

## Conclusions

The Chinese version of the TAS-20 was an acceptable instrument for evaluating this large sample of medical students in China. Gender, physical activity, grade, history of living with parents, and childhood trauma were all associated the level of alexithymia. Male medical students and medical students in the fifth grade experienced higher level of alexithymia. Less physical activity, living without parents during childhood and having childhood trauma were risk factors of alexithymia. To prevent alexithymia, it is necessary to promote adequate physical activity and to place greater emphasis on initiatives that target male medical students and those in the final year of training. The next step of research should be to carry out interventions on alexithymia in Chinese medical students, and to evaluate the intervention effectiveness. Additionally, we also suggest that the individual influencing factors that we have identified be addressed separately and in more depth in future research.

## Additional files

Additional file 1: 20-item Toronto Alexithymia Scale (TAS-20). (DOCX 15 kb)

## Abbreviations

CFA: Confirmatory factor analysis
DDF: Difficulty describing feelings
DIF: Difficulty identifying feelings
EOT: Externally oriented thinking
NMA: Normative male alexithymia.
TAS-20: 20-item Toronto Alexithymia Scale
