# Supplementary material for: Influencing factors of alexithymia in Chinese medical students: a cross-sectional study
Source: BMC Med Educ. 2017 Apr 4;17:66. doi: 10.1186/s12909-017-0901-8 (PMC5379661; doi:10.1186/s12909-017-0901-8)
Supplement: Supplementary file 1 — 20-item Toronto Alexithymia Scale (TAS-20). (DOCX 15 kb) [file 12909_2017_901_MOESM1_ESM.docx]

**The 20-item Toronto Alexithymia Scale-I**

*Please indicate the extent to which you agreed or disagreed with each statement.

|  | strongly agree | moderately agree | neither disagree or agree | moderately disagree | strongly disagree |
| --- | --- | --- | --- | --- | --- |
| 1. I am often confused about what emotion I am feeling. | 5 | 4 | 3 | 2 | 1 |
| 2. It is difficult for me to find the right words for my feelings. | 5 | 4 | 3 | 2 | 1 |
| 3. I have physical sensations that even doctors don't understand | 5 | 4 | 3 | 2 | 1 |
| 4. I am able to describe my feelings easily. | 5 | 4 | 3 | 2 | 1 |
| 5. I prefer to analyze problems rather than just describe them. | 5 | 4 | 3 | 2 | 1 |
| 6. When I am upset, I don't know if I am sad, frightened, or angry. | 5 | 4 | 3 | 2 | 1 |
| 7. I am often puzzled by sensations in my body. | 5 | 4 | 3 | 2 | 1 |
| 8. I prefer to just let things happen rather than to understand why they turned out that way. | 5 | 4 | 3 | 2 | 1 |
| 9. I have feelings that I can't quite identify. | 5 | 4 | 3 | 2 | 1 |
| 10. Being in touch with emotions is essential. | 5 | 4 | 3 | 2 | 1 |
| 11. I find it hard to describe how I feel about people. | 5 | 4 | 3 | 2 | 1 |
| 12. People tell me to describe my feelings more. | 5 | 4 | 3 | 2 | 1 |
| 13. I don't know what's going on inside me. | 5 | 4 | 3 | 2 | 1 |
| 14. I often don't know why I am angry. | 5 | 4 | 3 | 2 | 1 |
| 1. I prefer talking to people about their daily activities rather than their feelings. | 5 | 4 | 3 | 2 | 1 |
| 16. I prefer to watch "light" entertainment shows rather than psychological dramas. | 5 | 4 | 3 | 2 | 1 |
| 17. It is difficult for me to reveal my innermost feelings, even to close friends. | 5 | 4 | 3 | 2 | 1 |
| 18. I can feel close to someone, even in moments of silence. | 5 | 4 | 3 | 2 | 1 |
| 19. I find examination of my feelings useful in solving personal problems. | 5 | 4 | 3 | 2 | 1 |
| 20. Looking for hidden meanings in movies or plays distracts from their enjoyment. | 5 | 4 | 3 | 2 | 1 |
